# Editorial: Explicit and Implicit Emotion Processing: Neural Basis, Perceptual and Cognitive Mechanisms

**DOI:** 10.3389/fpsyg.2020.584469

**Published:** 2020-09-30

**Authors:** Alessia Celeghin, Noemi Mazzoni, Giulia Mattavelli

**Affiliations:** ^1^Department of Psychology, University of Torino, Torino, Italy; ^2^Department of Psychology and Cognitive Science, University of Trento, Trento, Italy; ^3^School of Advanced Studies Istituto Universitario Studi Superiori Pavia, Pavia, Italy

**Keywords:** emotion perception, implicit - explicit processing, emotional contagion, autism spectrum disorder (ASD), Parkinson disease, active inference, emotion regulation (ER)

The scientific construct of “emotion” has been characterized in the past century by a plethora of different properties (Mauss and Robinson, 2009; Fox, 2018). Understandably, research has emphasized the systematic decomposition of this complex subject of inquiry into elemental components. This approach made great strides in the understanding of key emotional components, and has been seminal in tackling neural fingerprint of emotion, its different behavioral responses, physiological reactions, motivational boosts, and communicative consequences. The investigation through separate and parallel channels represented the most effective way to build an operational scientific approach capable to provide the rigor, replicability, and proper control over the selected components. Nevertheless, each strategy presents some limitations: for example, neuroimaging studies offer correlational evidence about brain activity and the corresponding emotional response, while lesion studies offer causal evidence on altered mental processing, but scarce statistical reproducibility and issues related to homogeneity among groups of patients (Caramazza, 1986). It follows that the analysis of behavior integrated with more sophisticated methods is crucial to offer new perspectives on the emotion field (Krakauer et al., 2017).

A peculiar property of emotions consists in their capability to modulate human behavior both at explicit and implicit levels (Gyurak et al., 2011; Braunstein et al., 2017). The ongoing debate considers the type of and the way stimuli are presented, the definition of automaticity and its neural mechanism, the proper methods to collect those responses (Pourtois et al., 2013). This debate prompted the launch of the current Research Topic, which collects several articles and offers an updated view on the multiplicity of approaches in affective sciences. For instance, Burra and Kerzel emphasize the role of top-down processes and attentional demands in the perceptual encoding of emotions and the prioritization of threatening stimuli. Oldrati et al. also focus on the interplay between attention and emotion, and identify the contribution of stimulus type and intensity in summoning attentional resources. Besides attention, also social factors and internal states can modulate emotional encoding. The automatic transmission of emotional content from one individual to another one, the so-called emotional contagion, is a prominent example of reflex-like reactions that typifies initial stages of emotional responses, which are likely to have an evolutionary origin. Often described as a motor imitation (i.e., mimicry), it occurs in a way that is highly dependent on the individual's learning history, often insensitive to social context, although some rapid responses seem modulated by social factors (Hatfield et al., 1994). In their study, Norscia et al. investigate the possible relation between emotional contagion and (auditory) yawn contagion, a form of replication of facial display triggered by an auditory signal. The authors address the affiliation-based accounts (Palagi et al., 2020) and provide evidence supporting the contention that familiarity, gender bias, and social bond drive the rate and efficiency of contagion. The role of the contextual state in the judgment of emotional expression is also addressed by Pinilla et al., who investigate how an induced affective state can affect the judgment of the observed affective state in others, as communicated by face. Other relevant contextual elements are the mutual interactions amid visual perception of social cues to derive social categories, such as gender and race, and how these processes modulate emotion perception and guide interpersonal behaviors (Bagnis et al., 2019). These aspects are investigated by Venturoso et al. in their article describing how gender modulates the implicit processing of in-group and out-group faces, and by Vergallito et al., who describe how own vs. others expressions alter emotion perception at the sensorimotor level.

Clinical studies have been invaluable to reveal the causal contribution of specific brain areas in emotion processing. Indeed, deficits in the recognition of distinct emotions result from focal lesions in different brain areas, like the amygdala, anterior insula, basal ganglia and frontal cortex (Calder et al., 2001; Celeghin et al., 2017; Mattavelli et al., 2019). Likewise, patients with neurodegenerative diseases involving the insula and basal ganglia (such as Huntington's and Parkinson's diseases) showed poorer performance in emotion recognition, with possible dissociations related to the stages of processing (Novak et al., 2012; Mattavelli et al., 2020). In their review, Wagenbreth et al. focus on patients with Parkinson and deep brain stimulation (DBS) in the subthalamic nucleus. They conclude that DBS has no consistent impact on facial emotion recognition although improvements were measurable for non-facial signals—like written or spoken semantic stimuli and emotional prosody—and non-facial pictorial stimuli. Moreover, a limited number of studies showed less impairment in implicit processing associated with DBS. This is in line with the view of at least partially distinct pathways for explicit and implicit emotion processing, a perspective proposed also for accounting for emotion regulation dysfunctions in individuals with autism spectrum disorder (ASD). In this regard, Siciliano and Clausi outline the role of the cerebellum in integrating internal state with external environmental information. Two other studies in the Topic address emotional difficulties in ASD: Mazzoni et al. show that the difficulty in recognizing emotional body stimuli in children with ASD may rely on atypical processing of body movement information rather than on emotion itself; Giannotti et al. find that alexithymia rather than ASD predicts perception of attachment to parents.

The hierarchical integration of high and low levels of emotional encoding—and, among these, the interoceptive and exteroceptive embodied cues—is a crucial topic in understanding emotion regulation. In this regard, the perspective discussed by Shalev conceptualizes low emotional clarity as the product of low access to the emotional signal. This results in prediction error associated with a mismatch between the current bodily state and the predicted state. The motivated cue integration theory (MCI) proposed by the author describes the potential transformation of low emotional clarity into the creation of emotion goals. Within this frame, a special emphasis has been observed in deep generative models that afford the capacity to explain multimodal (i.e., interoceptive, proprioceptive, and exteroceptive) sensations that are characteristic of emotion concepts. For instance, Smith et al. introduce a novel model of emotion inference that is sufficiently nuanced to produce synthetic emotional processes. Their computational simulation infers the emotion by considering the feeling in a specific moment and conceptualizing the learning process. Interestingly, they outline an active inference model of emotional states in the brain, which can minimize uncertainty to maximize prior preferences. This model—described by the Bayesian principles—postulates that prior information could influence the interpretation of sensory evidence, especially when this latter is ambiguous or degraded. As it happens, prior probabilities could be represented by automatically driven psychophysiological changes that influence perceptual decisions on emotions. From these pieces of evidence, a new avenue to implement a Bayesian framework in the domain of non-conscious and conscious emotional perception seems an interesting step to follow.

In conclusion, with this Topic, we sought to offer a comprehensive picture of a phenomenon as multifaceted as explicit and implicit emotion processing is. The variety of studies included here well pictures the current research efforts to integrate different points of view. The future challenge is to converge into a comprehensive theoretical model of the emotional brain, taking into account multiple factors that require a more conjoint and complementary form of integration.

## Author Contributions

All authors listed have made a substantial, direct and intellectual contribution to the work, and approved it for publication.

## Conflict of Interest

The authors declare that the research was conducted in the absence of any commercial or financial relationships that could be construed as a potential conflict of interest.

## References

[B1] Bagnis A. Celeghin A. Mosso C. O. Tamietto M. (2019). Toward an integrative science of social vision in intergroup bias. Neurosci. Biobehav. Rev. 102, 318–326. 10.1016/j.neubiorev.2019.04.020 31042557

[B2] Braunstein L. M. Gross J. J. Ochsner K. N. (2017). Explicit and implicit emotion regulation: a multi-level framework. Soc. Cogn. Affect. Neurosci. 12, 1545–1557. 10.1093/scan/nsx096 28981910 PMC5647798

[B3] Calder A. J. Lawrence A. D. Young A. W. (2001). Neuropsychology of fear and loathing. Nat. Rev. Neurosci. 2, 352–363. 10.1038/35072584 11331919

[B4] Caramazza A. (1986). On drawing inferences about the structure of normal cognitive systems from the analysis of patterns of impaired performance: the case for single-patient studies. Brain Cogn. 5, 41–66. 10.1016/0278-2626(86)90061-8 3954906

[B5] Celeghin A. Diano M. Bagnis A. Viola M. Tamietto M. (2017). Basic emotions in human neuroscience: neuroimaging and beyond. Front. Psychol. 8:1432. 10.3389/fpsyg.2017.01432 28883803 PMC5573709

[B6] Fox E. (2018). Perspectives from affective science on understanding the nature of emotion. Brain Neurosci. Adv. 2:2398212818812628. 10.1177/2398212818812628 32166161 PMC7058241

[B7] Gyurak A. Gross J. J. Etkin A. (2011). Explicit and implicit emotion regulation: a dual-process framework. Cogn. Emot. 25, 400–412. 10.1080/02699931.2010.544160 21432682 PMC3280343

[B8] Hatfield E. Cacioppo J. T. Rapson R. L. (1994). Emotional Contagion. Cambridge: Cambridge University Press 10.1017/CBO9781139174138

[B9] Krakauer J. W. Ghazanfar A. A. Gomez-Marin A. MacIver M. A. Poeppel D. (2017). Neuroscience needs behavior: correcting a reductionist bias. Neuron 93, 480–490. 10.1016/j.neuron.2016.12.041 28182904

[B10] Mattavelli G. Barvas E. Longo C. Zappini F. Ottaviani D. Malaguti M. C.. (2020). Facial expressions recognition and discrimination in Parkinson' s disease. J. Neuropsychol.. 10.1111/jnp.12209 32319735

[B11] Mattavelli G. Pisoni A. Casarotti A. Comi A. Sera G. Riva M.. (2019). Consequences of brain tumour resection on emotion recognition. J. Neuropsychol. 13, 1–21. 10.1111/jnp.12130 28700143

[B12] Mauss I. B. Robinson M. D. (2009). Measures of emotion: a review. Cogn. Emot. 23, 209–237. 10.1080/02699930802204677 19809584 PMC2756702

[B13] Novak M. J. U. Warren J. D. Henley S. M. D. Draganski B. Frackowiak R. S. Tabrizi S. J. (2012). Altered brain mechanisms of emotion processing in pre-manifest Huntington's disease. Brain 135, 1165–1179. 10.1093/brain/aws024 22505631 PMC3326253

[B14] Palagi E. Celeghin A. Tamietto M. Winkielman P. Norscia I. (2020). The neuroethology of spontaneous mimicry and emotional contagion in human and non-human animals. Neurosci. Biobehav. Rev. 111, 149–165. 10.1016/j.neubiorev.2020.01.020 31972204

[B15] Pourtois G. Schettino A. Vuilleumier P. (2013). Brain mechanisms for emotional influences on perception and attention: what is magic and what is not. Biol. Psychol. 92, 492–512. 10.1016/j.biopsycho.2012.02.007 22373657

